# Decreased expression of GRIM-19 by DNA hypermethylation promotes aerobic glycolysis and cell proliferation in head and neck squamous cell carcinoma

**DOI:** 10.18632/oncotarget.2684

**Published:** 2014-12-23

**Authors:** Xiao-Yun Zhang, Minle Li, Kai Sun, Xiao-Jie Chen, Jian Meng, Lifang Wu, Ping Zhang, Xuemei Tong, Wei-Wen Jiang

**Affiliations:** ^1^ Department of Oral Mucosal Diseases, Shanghai Ninth People's Hospital, Shanghai Jiao Tong University School of Medicine, Shanghai 200011, China; ^2^ Department of Biochemistry and Molecular Cell Biology, Shanghai Key Laboratory for Tumor Microenvironment and Inflammation, Shanghai Jiao Tong University School of Medicine, Shanghai 200025, China

**Keywords:** GRIM-19, methylation, HNSCC, proliferation, metabolism

## Abstract

To identify novel tumor suppressor genes that are down-regulated by promoter hypermethylation in head and neck squamous cell carcinoma (HNSCC), genome-wide methylation profiling was performed using a methylated DNA immunoprecipitation (MeDIP) array in HNSCC and normal mucosa tissue samples. Promoter hypermethylation of the candidate gene, gene associated with retinoid-interferon induced mortality-19 (GRIM-19), was confirmed in HNSCC cell lines. Multivariate regression analysis determined that GRIM-19 hypermethylation was an independent significant factor for HNSCC diagnosis (OR:125.562; *P* < 0.001). HNSCC patients with lower ratio of GRIM-19/ACTB hypermethylation had increased overall and disease free survival. Furthermore, the optimal cutoff provided 90% sensitivity and 77% specificity of GRIM-19 hypermethylation as a diagnostic marker for HNSCC. Ectopic expression of GRIM-19 in HNSCC cells led to increased oxygen consumption, reduced glycolysis and decreased cell proliferation. HNSCC cells ectopically expressing GRIM-19 displayed increased p53 activity as well as decreased Stat3 and HIF-1α activities. Moreover, GRIM-19 knockdown not only resulted in decreased oxygen consumption and increased aerobic glycolysis but also promoted cell proliferation and tumorigenic capacity in HNSCC cells. Our data indicate that decreased GRIM-19 expression due to promoter hypermethylation may be important in head and neck carcinogenesis by promoting cell proliferation and regulating metabolic activity.

## INTRODUCTION

Head and neck squamous cell carcinoma (HNSCC) is one of the most common cancer, accounting for over 650,000 new cases and 350,000 cancer deaths every year worldwide [1–3] and HNSCC incidence increases with age. Tobacco smoking, alcohol consumption, environmental exposures, and HPV infection have been identified as important risk factors for HNSCC development. Although there have been improvements in surgery, radiotherapy, and chemotherapy, the 5-year survival for advanced HNSCC remains low. Many efforts have been attempted to identify molecular events that occur during HNSCC development, including the inactivation of TP53, Notch mutations [4, 5], and altered metabolites [6]. Further elucidation of the molecular mechanisms in head and neck carcinogenesis are expected to accelerate the development of efficacious anticancer agents and the identification of diagnostic or therapeutic biomarkers.

DNA methylation is associated with a number of key processes, including embryonic development, X chromosome inactivation, and genomic imprinting. Aberrant DNA methylation has been observed in many cancers [7]. However, the role of DNA methylation in HNSCC initiation and progression remains largely unknown [8].

Gene associated with retinoid-interferon induced mortality-19 (GRIM-19) encodes a subunit of the complex I of mitochondrial membrane respiratory chain and is present in both the nuclear and cytoplasmic cellular compartments. GRIM-19 protein is required for electron transfer activity and complex I assembly [9]. GRIM-19 overexpression induces apoptosis in breast, prostate, and renal carcinoma cells [9]. GRIM-19 binds the Stat3 transcription factor and inhibits Stat3 activity [10]. Altered expression and mutation of GRIM-19 has been observed in various tumors including lung cancer [11], hepatocellular carcinoma (HCC) [12], breast cancer [13], glioma [14], renal cell carcinoma (RCC) [15] and HNSCC [16]. However, the underlying mechanism of GRIM-19 down-regulation remains unclear.

In this study, we used methylated DNA immunoprecipitation (MeDIP) to investigate the whole-genome distribution of aberrant DNA methylation in tissue samples from HNSCC and the oral mucosa of healthy subjects, to identify novel hypermethylated candidate genes in HNSCC. Methylated CpG islands (CGIs) were widespread in the gene-related regions, in which 3034 for normal and 4669 for HNSCC, respectively, and in the genomic unknown regions, in which 1416 for normal and 2376 for HNSCC, respectively. We validated the hypermethylation status of a representative candidate, GRIM-19, in patients with HNSCC. Furthermore, we observed that altered GRIM-19 expression not only regulated p53 and HIF-1α activities, but also altered aerobic glycolysis, cell proliferation and tumorigenic capacity of HNSCC cells. Our results indicate that GRIM-19 hypermethylation may serve as a biomarker in HNSCC.

## RESULTS

### Genomic distribution of DNA methylation in HNSCC and normal mucosa

**Figure 1:**
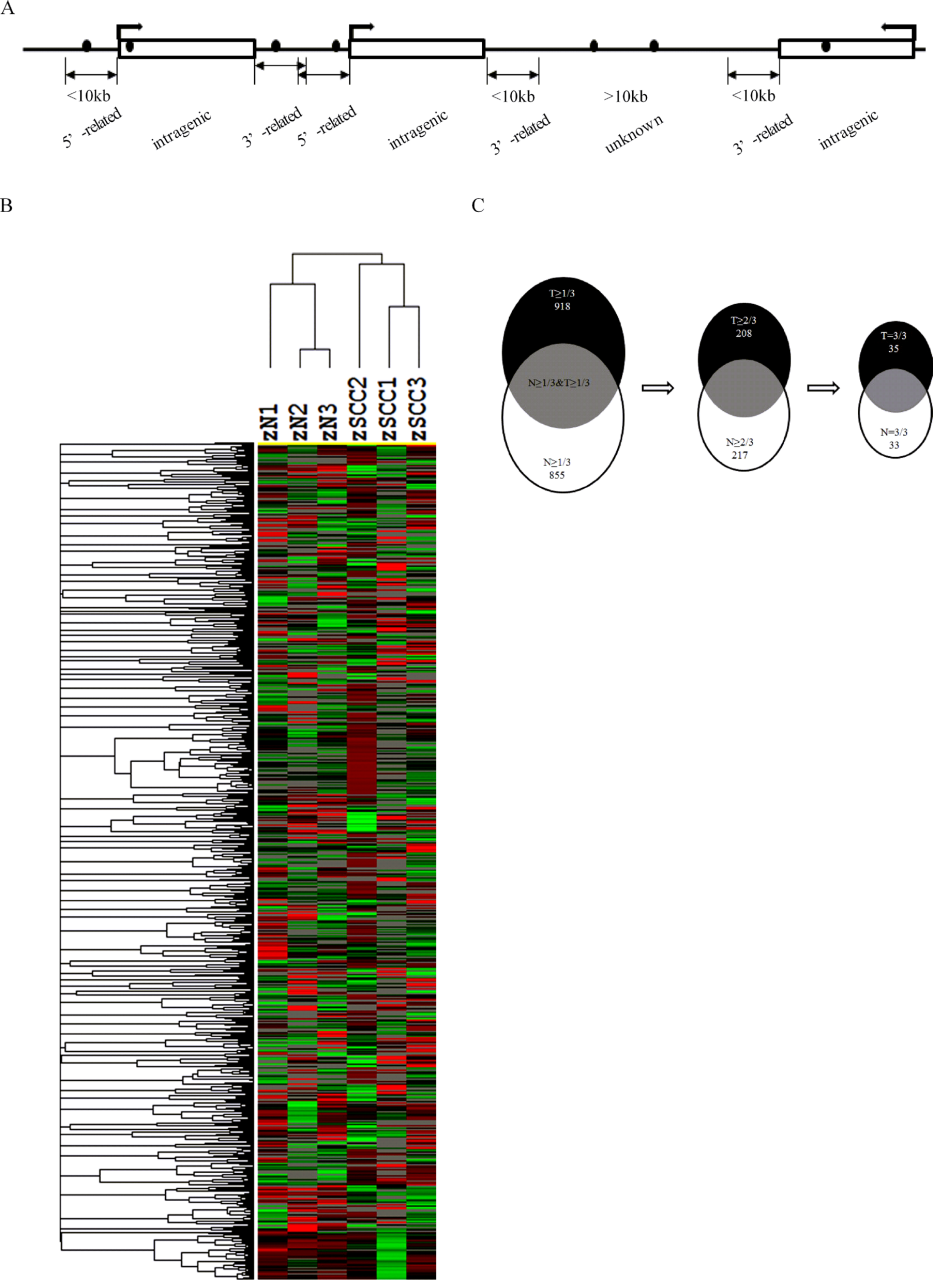
Genome-wide DNA methylation profile and screening for altered DNA methylation candidate genes in HNSCC **(A)** Diagram of DNA methylation sites in genome. Black dot, methylated CGI; swerved arrow, direction of transcription; rectangle, a gene; 5′-related, 5′-gene related region; 3′-related, 3′-gene related region; intragenic, intragenic region; unkown, genomic unknown regions without any gene annotations. **(B)** Two-dimensional hierarchical clustering in HNSCC and control samples. Probes are in rows and samples are in columns. **(C)** Outline of screening strategy for candidate genes with altered promoter methylation. T, HNSCC; N, normal control.

DNA methylation has been associated with genome regulation in cancers and normal tissues. To better understand global changes of DNA methylation, genome-wide methylation was examined in both 3 HNSCC tissues and 3 normal mucosa. We observed 2202 methylated CGIs of 7045 (31%) in HNSCC and 1787 of 4450 (40%) in normal mucosa in the 5′-gene related region, (Figure 1A and Figure S1A). Only 1082 of 7045 (15%) in HNSCC and 457 of 4450 (10%) methylated CGIs in normal mucosa were observed in 3′-gene related region. Overall, there were 66% and 68% methylated CGIs located within or near genes. Intriguingly, 34% and 32% of the methylated CGIs were mapped to genomic unknown regions that lack any gene annotation in HNSCC and normal mucosa, respectively (Figure 1A and Figure S1A).

Aberrant hypermethylation in gene promoters has frequently been associated with many cancers types. Among the 2202 methylated CGIs identified in the 5′-gene related region for HNSCC, 81% (1793) methylated CGIs were within +/−1 kb from the transcription start site (TSS) (Figure S1B).

### Identification of aberrantly methylated candidate genes in HNSCC

DNA hypermethylation has emerged as sensitive and specific biomarkers in many cancers. We assessed if the differentially methylated CGIs identified in current study could effectively distinguish HNSCC from normal oral mucosa. Figure 1B shows the unsupervised hierarchical clustering of the DNA methylation of normal mucosa compared with HNSCC. The normal and HNSCC samples are correctly classified. Furthermore, 1792 identified methylated CGIs overlapped with 1762 gene promoters. Among these promoters, 918 genes promoters were hypermethylated in at least 1 out of 3 (33%) HNSCC samples, but they were negative in the normal mucosa samples. Because their promoters were hypermethylated in all HNSCC samples but not in normal mucosa samples, 35 genes were defined as highly hypermethylated candidates (Figure 1C). A number of these highly hypermethylated candidates, including SOX1 [17–20], WIF1 [21, 22], ACRC [23], ADCY5 [24], BCAP31 [25], NKX6-2 [26], PLK5P [27], and PRSS21 [28], have previously been reported as promoter hypermethylated genes in various cancers (Table 1). We also identified novel highly hypermethylated genes such as GRIM-19 and ABCD1, which have previously been reported to have altered expressions in cancers [11–14, 29] (Table 2). To further characterize the candidate genes regulated by DNA methylation, we used the KEGG Orthology annotation program to group genes according to their biological functions. Among the hypermethylated candidate genes, six of them including ROCK2, PRKACG, FRAT1, SMAD3, TBL1X, and WIF1 are members of the Wnt signaling pathway (Table 1 and Table S1), which suggests that underexpression of these genes may contribute to head and neck carcinogenesis. In addition, we identified 33 genes that were frequently hypomethylated in the HNSCCs compared with normal mucosa (Table S2), which suggested that hypomethylation mediated activation of these genes may be involved in HNSCC development.

**Table 1:** Highly hypermethylated candidate genes in HNSCC

Gene	Methylation reported in tumor	Decreased expression in tumor	KEGG Pathway
ABCD1		Renal CA^30^	Peroxisome
ACRC	Ewing sarcoma^23^		
ADCY5	Lung adenocarcinoma^24^		Gap junction, Dilated cardiomyopathy
ADM2			
ATG10			
B3GNT9			
BCAP31	Breast CA^25^		
CCNY			
CLCN7			
FAM148C			
FGD1			
FJX1			
GGN			
KCNJ12			
MAP7D3			
MBP			
MKL1			
MMGT1			
GRIM19		Lung CA^11^, Liver CA^12^, Breast CA^13^, Glioma^14^	
NKX6-2	Lung adenocarcinoma^24^		
NUDT19			Peroxisome
PAQR4			
PLK5P	Glioblastoma^27^		
PRSS21	Testicular^28^		
PTF1A			
PTPN20B			
SELO			
SOX1	Oral CA^17^, Cervical CA^18^, Ovarian CA^19^, Prostate CA^20^		
STK25			
TSPYL2			
VMA21			
WDR40B			
WDR40C			
WIF1	Oral CA^21^, Colon CA^22^		Wnt signaling pathway
YJEFN3			

CA, carcinoma.

**Table 2:** Clinical features of HNSCC and control

	HNSCC	Control
**Number of subjects**	30	31
**Mean age (years, range)**	54 (28-79)	43 (23-69)
**Age (%)**		
≤ 55	15 (50%)	24 (77%)
> 55	15 (50%)	7 (23%)
**Gender (%)**		
Male	13 (43%)	13 (42%)
Female	17 (57%)	18 (58%)
**TMN ^a^ stage**		
**T (%)**		
T 1	6 (20%)	
T 2	9 (30%)	
T 3	9 (30%)	
T 4	4 (13%)	
NA	2 (7%)	
**M (%)**		
M 0	30 (100%)	
**N (%)**		
N 0	19 (63%)	
N 1	6 (20%)	
N 2	3 (10%)	
NA	2 (7%)	
**Smoking (%)**		
Never	21 (70%)	11 (35%)
Past	2 (7%)	1 (3%)
Current	7 (23%)	3 (10%)
NA	0 (0%)	16 (52%)

a Union for International Cancer Control; T, tumor size; N, lymph node; M, Metastasis; NA, data not available; Never, no smoking history; Past, stop smoking over one year. Current, current smoker.

### Verification of GRIM-19 promoter hypermethylation in HNSCC

**Figure 2:**
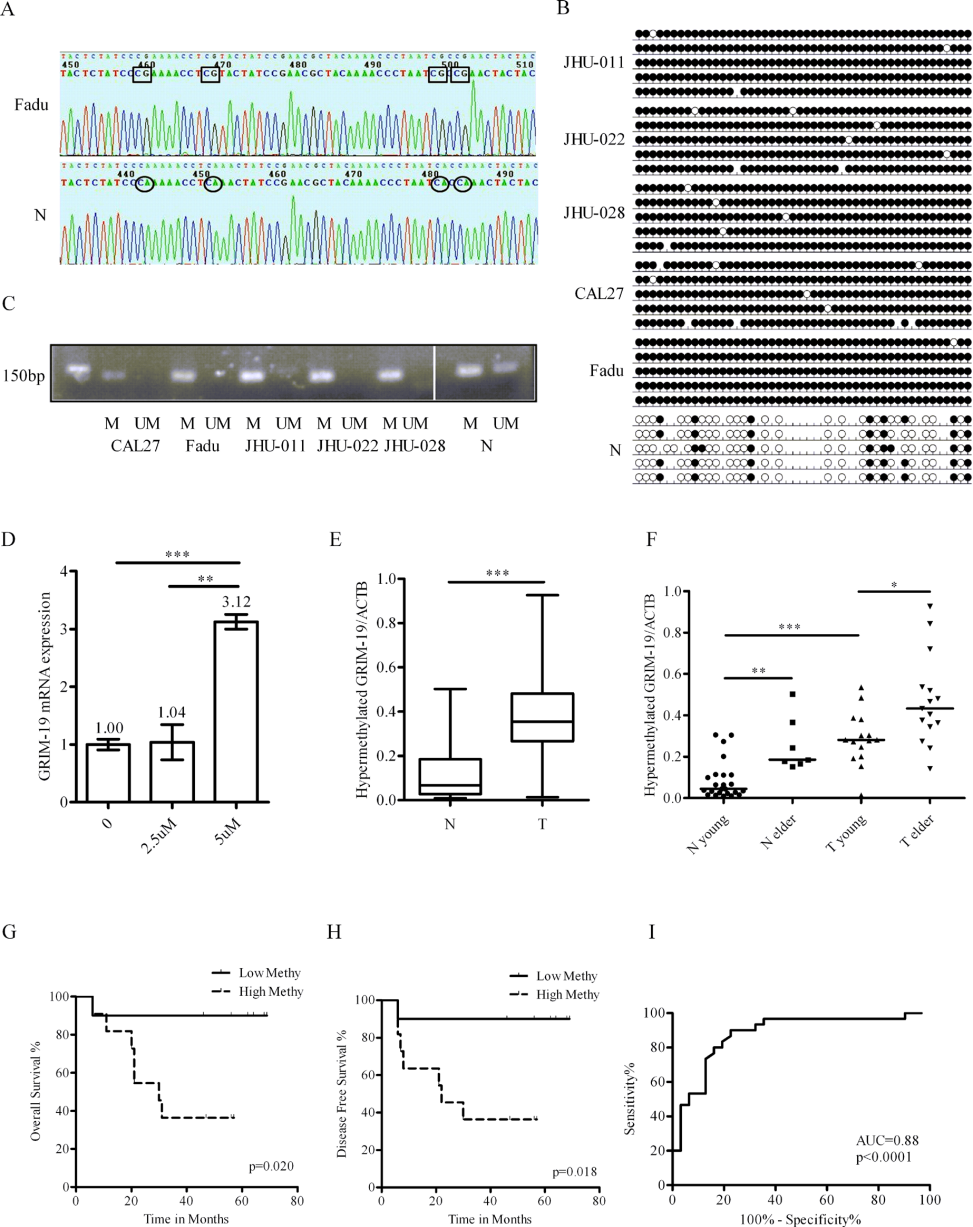
Verification and clinical significance of GRIM-19 hypermethylation **(A)** Representative sequences of BSP in Fadu cell line and normal mucosa. Rectangle, sequence of methylated CG; circle, sequence of unmethylated CG. **(B)** Dot graph of BSP data in HNSCC cell lines and normal mucosa. Black dot, methylated CG; white dot, unmethylated CG; stub, not available. **(C)** MSP of GRIM-19 in HNSCC cell lines and normal mucosa. **(D)** RT-QPCR analysis of GRIM-19 mRNA expression after 0, 2.5 or 5 μM 5-AZA treatment for 24 hours. **P < 0.01; ***P < 0.001. **(E)** Comparison of GRIM-19 hypermethylation between HNSCC and normal by QMSP. N, normal; T, HNSCC. **(F)** Comparison of GRIM-19 hypermethylation between young and elder subjects by QMSP. *P < 0.05. **(G)** The overall survival analysis of HNSCC with lower or higher GRIM-19 methylation by Log-Rank test. Low Methy, HNSCC with lower GRIM-19 methylation; High Methy, HNSCC with higher GRIM-19 methylation. **(H)** The disease free survival analysis of HNSCC with lower or higher GRIM-19 methylation by Log-Rank test. **(I)** The sensitivity and specificity of GRIM-19 hypermethylation by ROC analysis.

The promoter hypermethylation status of GRIM-19 in HNSCC and normal controls was verified using bisulfate sequencing PCR (BSP) (Figure 2A, B) and methylation-specific PCR (MSP) (Figure 2C) analyses. We observed that GRIM-19 was hypermethylated as predicted in HNSCC lines, but only partially methylated in normal controls (Figure 2B, C). To assess if a demethylation agent could restore transcriptional activity, CAL27 cells were treated with 2.5 or 5 μM 5-AZA for 24 and 48 h. The mRNA expression of GRIM-19 was increased after 5-AZA treatment (Figure 2D), but it was not quantity- or time- dependent (data not shown).

### Clinical significance of GRIM-19 promoter hypermethylation in HNSCC

To determine if GRIM-19 is hypermethylated in primary HNSCC in a tumor-specific manner, a new cohort of 30 HNSCC and 31 normal mucosa samples was analyzed using QMSP. The relevant clinicopathologic parameters of the 61 subjects are summarized in Table 1. Our data demonstrated that the median level of GRIM-19 methylation in HNSCC (0.354) was significantly higher compared to normal mucosa tissues (0.067; Mann-Whitney test, *P* < 0.001) (Figure 2E). The GRIM-19 mRNA expression tended to be lower in HNSCC, but the difference was not statistically significant (data not shown). We further sub-grouped subjects into young (≤ 55 years) and elderly (> 55) groups. In both HNSCC and normal samples, elderly subjects had higher hypermethylation levels than younger subjects (Figure 2F). A multivariate regression model analysis revealed that HNSCC diagnosis (OR: 32.275; *P* = 0.005) and age (OR: 1.163; *P* = 0.001) were independent risk factors for GRIM-19 hypermethylation. Tumor site, stage, gender, smoking or alcohol consumption was not found to affect GRIM-19 hypermethylation (*P* > 0.05). However, only GRIM-19 hypermethylation was an independent risk factor for HNSCC diagnosis. As the ratio of GRIM-19/ACTB hypermethylation increased by 0.001 increments, the risk for HNSCC increased 125.562-fold (*P* < 0.001). Furthermore, HNSCC patients with a lower ratio of GRIM-19/ACTB hypermethylation were observed to have improved overall survival and disease free survival (Figure 2G, H). To determine the appropriate cutoff for a potential biomarker application, we performed an ROC analysis. The area under ROC (AUC) was 0.88 (*P* < 0.0001). The optimal cutoff, as defined by Youden's index, provided 90% sensitivity and 77% specificity for GRIM-19 hypermethylation status as a diagnosis marker for HNSCC (Figure 2I).

### Glucose and oxygen consumption correlates with GRIM-19 expression in HNSCC cell lines

**Figure 3:**
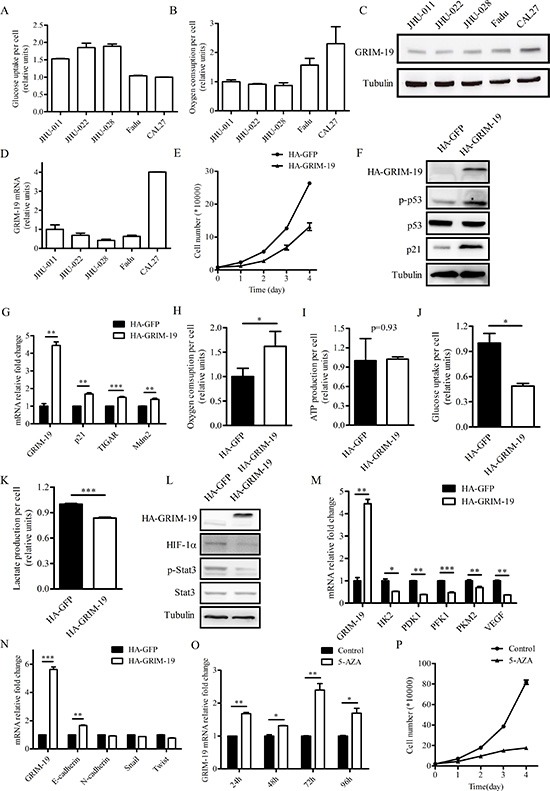
Ectopically expressed GRIM-19 increases oxygen consumption and decreases cell proliferation in JHU-028 cells **(A)** Glucose uptake and **(B)** oxygen consumption of five HNSCC cell lines. The data in (A) and (B) were normalized by cell number. **(C)** Western blot analysis of protein extracts of five HNSCC cell lines using antibodies to GRIM-19 and tubulin. **(D)** QPCR analysis of endogenous GRIM-19 mRNA in HNSCC cell lines. **(E)** Cell proliferation of JHU-028 cells stably expressing either HA-GFP or HA-GRIM-19. **(F)** Western blot analysis of protein extracts of JHU-028 cells stably expressing either HA-GFP or HA-GRIM-19 using antibodies to HA, p-p53, p53, p21 and tubulin. **(G)** QPCR analysis of GRIM-19, p21, TIGAR, and Mdm2 in JHU-028 cells stably expressing either HA-GFP or HA-GRIM-19. **(H)** Oxygen consumption, **(I)** ATP production, **(J)** glucose uptake, and **(K)** lactate production of JHU-028 cells stably expressing either HA-GFP or HA-GRIM-19. **(L)** Western blot analysis of protein extracts of JHU-028 cells stably expressing either HA-GFP or HA-GRIM-19 using HIF-1α, p-Stat3, Stat3 and tubulin antibodies. **(M)** QPCR analysis of GRIM-19, HK2, PDK1, PFK1, PKM2 and VEGF in JHU-028 cells stably expressing either HA-GFP or HA-GRIM-19. **(N)** QPCR analysis of GRIM-19, E-cadherin, N-cadherin, Snail and Twist in JHU-028 cells stably expressing either HA-GFP or HA-GRIM-19. **(O)** QPCR analysis of endogenous GRIM-19 mRNA in JHU-028 cell lines at 24, 48, 72 and 96 hours after vehicle control or 5 μM 5-AZA treatment. **(P)** Cell proliferation of JHU-028 cells after vehicle control or 5 μM 5-AZA treatment. Tubulin blots in (C), (F) and (L) serve as loading controls.

To investigate the metabolic activities of different HNSCC cell lines, we compared the glucose uptake and oxygen consumption of JHU-011, JHU-022, JHU-028, Fadu and CAL27 cells. Fadu and CAL27 cells exhibited lower amounts of glucose uptake per cell and higher rates of oxygen consumption per cell compared with JHU-011, JHU-022 and JHU-028 cells (Figure 3A, B). Next, we examined GRIM-19 protein and mRNA expression in JHU-011, JHU-022, JHU-028, Fadu and CAL27 cells (Figure 3C, D). We observed that GRIM-19 expression in HNSCC cell lines was positively and negatively correlated with oxygen consumption rate and glycolytic activity, respectively. This result suggests that GRIM-19 level may be related to the metabolic activity of HNSCC cells. We decided to choose JHU-028 and CAL27 cells, which had low and high levels of endogenous GRIM-19, respectively, for further GRIM-19 overexpression and knockdown studies.

### Ectopic GRIM-19 expression leads to a metabolic switch from aerobic glycolysis to mitochondrial respiration in JHU-028 cells

To determine if increased GRIM-19 expression alters the metabolic and proliferative activity of HNSCC cells, we generated stable JHU-028 cells that overexpressed either HA-tagged GFP cDNA (HA-GFP) or HA-tagged GRIM-19 cDNA (HA-GRIM-19). To observe the effects of GRIM-19 on cancer cell proliferation, we plated JHU-028 cells stably expressing either HA-GFP or HA-GRIM-19 at equal numbers and counted cell numbers at day 1, 2, 3 and 4 after plating. Cell proliferation was impaired in JHU-028 cells stably expressing HA-GRIM-19 compared to control cells (Figure 3E). We did not observe a difference in cell viability between JHU-028 cells stably expressing HA-GRIM-19 and control cells (data not shown). We observed that levels of both p53 phosphorylation (Ser-15) and p21 increased in JHU-028 cells stably expressing HA-GRIM-19 (Figure 3F). Moreover, the mRNA levels of several p53 target genes such as p21, TIGAR and Mdm2 increased when GRIM-19 was overexpressed in JHU-028 cells (Figure 3G). These results indicate that p53 is activated when GRIM-19 expression increases.

Because GRIM-19 is critical for the function of the electron transport chain and oxidative phosphorylation in mitochondria, we next examined the effect of ectopic GRIM-19 expression on oxygen consumption in JHU-028 cells. JHU-028 cells stably expressing HA-GRIM-19 displayed higher rate of oxygen consumption per cell compared to control cells (Figure 3H). However, increased oxygen consumption in JHU-028 cells stably expressing HA-GRIM-19 did not alter ATP levels (Figure 3I), which indicates that the activity of other ATP-producing metabolic pathways may be reduced in these cells. We compared the glycolytic activity of JHU-028 cells stably expressing either HA-GFP or HA-GRIM-19 by quantifying glucose uptake and lactate production per cell (Figure 3J, K). JHU-028 cells stably expressing HA-GRIM-19 displayed reduced glucose uptake and lactate production, indicating a loss of aerobic glycolysis. We observed that levels of both phosphorylated Stat3 and HIF-1α decreased in JHU-028 cells stably expressing HA-GRIM-19 (Figure 3L). Moreover, the mRNA level of several HIF-1α target genes such as HK2, PDK1, PFK1, PKM2 and VEGF decreased when GRIM-19 was overexpressed in JHU-028 cells (Figure 3M).

Stat3 has been reported to regulate epithelial-mesenchymal transition (EMT) of HNSCC [30]. Therefore, we examined mRNA levels of several EMT marker genes in JHU-028 cells stably expressing HA-GFP or HA-GRIM-19. These EMT markers include E-cadherin which is downregulated during EMT as well as N-cadherin, Snail and Twist which are often upregulated during the process [31]. JHU-028 cells ectopically expressing GRIM-19 showed higher E-cadherin mRNA levels compared with control cells (Figure 3N). Ectopic GRIM-19 expression seemed to have mild effect on mRNA expression of N-cadherin, Snail and Twist in JHU-028 cells (Figure 3N).

Since 5-AZA treatment was able to increase GRIM-19 mRNA expression (Figure 2D), we treated JHU-028 cells with control or 5 μM 5-AZA for 24, 48, 72 and 96 hours, respectively and assayed for GRIM-19 mRNA expression, cell proliferation and metabolic activities. As expected, JHU-028 cells treated with 5-AZA showed increased GRIM-19 mRNA levels compared with control-treated cells (Figure 3O). Similar to what we observed in JHU-028 cells overexpressing GRIM-19 (Figure 3E and 3H), 5-AZA treatment decreased cell proliferation and promoted oxygen consumption in JHU-028 cells (Figure 3P and Figure S2A). However, JHU-028 cells treated with 5-AZA displayed higher rates of glucose uptake and lactate production compared with control cells (Figure S2B and S2C), which is probably due to the demethylation effect of 5-AZA on other genes directly or indirectly regulating glycolysis.

### Suppression of GRIM-19 increases aerobic glycolysis, cell proliferation and tumorigenic capacity of CAL27 cells

**Figure 4:**
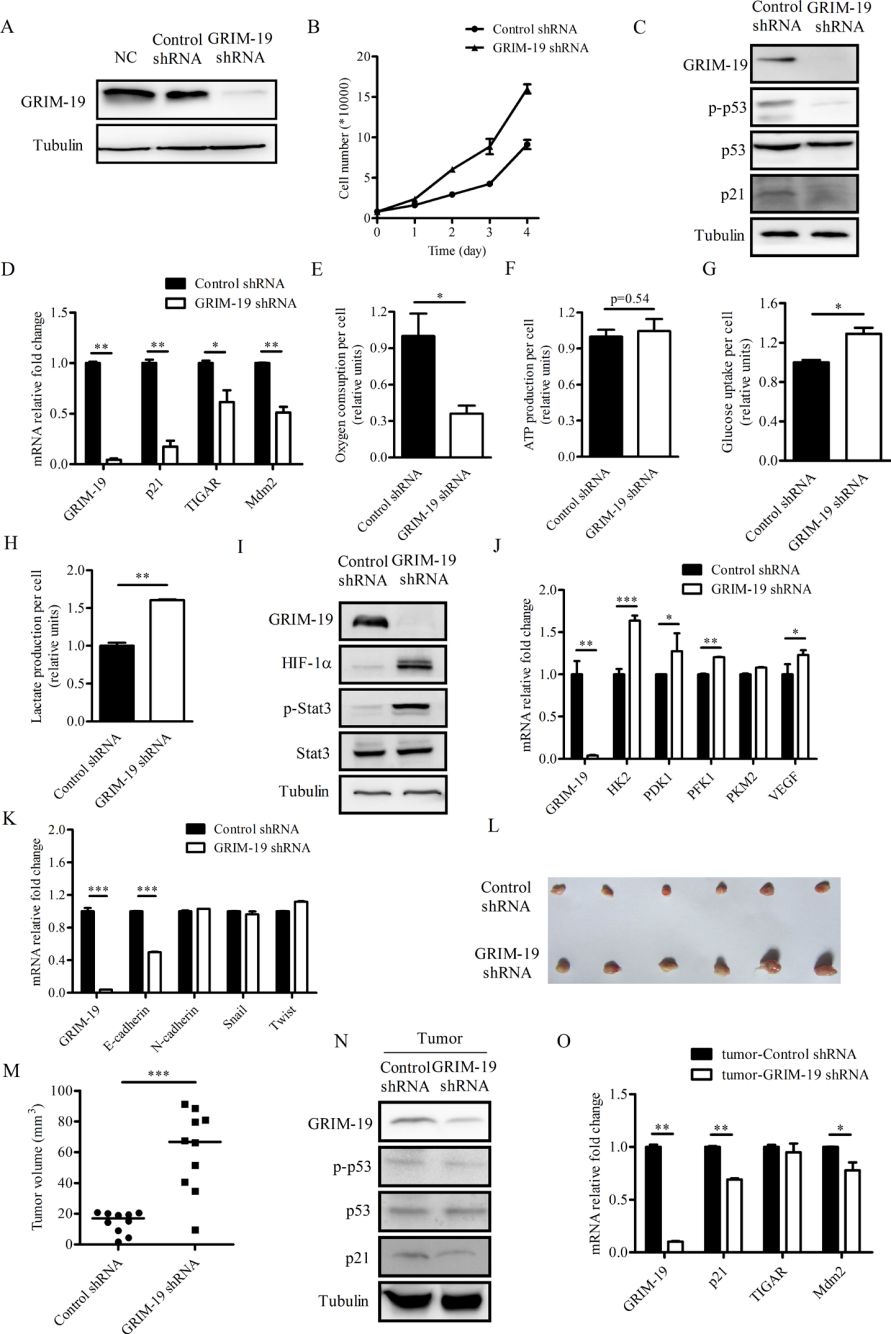
Suppression of GRIM-19 leads to decreased oxygen consumption, increased cell proliferation and tumorigenic capacity in CAL27 cells **(A)** Western blot analysis of protein extracts of parental CAL27 cells (NC) and CAL27 cells stably expressing either control or GRIM-19 shRNA using antibodies to GRIM-19 and tubulin. **(B)** Cell proliferation of CAL27 cells stably expressing either control or GRIM-19 shRNA. **(C)** Western blot analysis of GRIM-19, p-p53, p53, p21 and tubulin in the protein extracts of CAL27 cells stably expressing either control or GRIM-19 shRNA. **(D)** QPCR analysis of expression of GRIM-19, p21, TIGAR, and Mdm2 in CAL27 cells stably expressing either control or GRIM-19 shRNA. **(E)** Oxygen consumption, **(F)** ATP production, **(G)** glucose uptake and **(H)** lactate production of CAL27 cells stably expressing either control or GRIM-19 shRNA. The data in (E) - (H) are normalized by cell number. **(I)** Western blot analysis of GRIM-19, HIF-1α, p-Stat3, Stat3 and tubulin in the protein extracts of CAL27 cells stably expressing either control or GRIM-19 shRNA. **(J)** QPCR analysis of GRIM-19, HK2, PDK1, PFK1, PKM2 and VEGF in CAL27 cells stably expressing either control or GRIM-19 shRNA. **(K)** QPCR analysis of GRIM-19, E-cadherin, N-cadherin, Snail and Twist in CAL27 cells stably expressing either control or GRIM-19 shRNA. **(L)** Dissected tumors and **(M)** the mass of tumors from 10 nude mice 21 days after injection of CAL27 cells stably expressing either control or GRIM-19 shRNA. **(N)** Western blot analysis of protein extracts and **(O)** QPCR analysis of tumors formed in #1 nude mice 21 days after injection of CAL27 cells stably expressing either control or GRIM-19 shRNA. Western blot analysis of protein extracts of tumors formed in other nude mice were summarized in Figure S3. Tubulin blots in (A), (C), (I) and (N) serve as loading controls.

To determine the effects of stable GRIM-19 gene knockdown on the metabolic and proliferative activity of HNSCC cells, we established stable CAL27 cells that expressed either control shRNA or GRIM-19 shRNA. Stable expression of GRIM-19 shRNA led to an obvious decline in GRIM-19 protein levels (Figure 4A). To observe the effects of GRIM-19 knockdown on cancer cell proliferation, we plated CAL27 cells stably expressing either control shRNA or GRIM-19 shRNA at equal numbers and counted cell numbers at day 1, 2, 3 and 4 after plating. Cell proliferation increased in CAL27 cells stably expressing GRIM-19 shRNA compared to control cells (Figure 4B). The levels of both p53 phosphorylation (Ser-15) and p21 decreased in CAL27 cells stably expressing GRIM-19 shRNA (Figure 4C). Moreover, the mRNA levels of several p53 target genes such as p21, TIGAR and Mdm2 decreased when GRIM-19 expression was suppressed in CAL27 cells (Figure 4D).

We next examined the effect of GRIM-19 suppression on oxygen consumption in CAL27 cells. CAL27 cells stably expressing GRIM-19 shRNA displayed lower rate of oxygen consumption per cell compared to control cells (Figure 4E). However, decreased oxygen consumption in CAL27 cells stably expressing GRIM-19 shRNA did not alter ATP levels (Figure 4F). Moreover, CAL27 cells stably expressing GRIM-19 shRNA displayed increased glucose uptake and lactate production (Figure 4G, H), thereby, indicating higher aerobic glycolytic activity. Levels of both phosphorylated Stat3 and HIF-1α increased in CAL27 cells stably expressing GRIM-19 shRNA (Figure 4I). Moreover, the mRNA level of several HIF-1α target genes such as HK2, PDK1, PFK1, PKM2 and VEGF increased when GRIM-19 was suppressed in CAL27 cells (Figure 4J). We also examined mRNA levels of EMT marker genes including E-cadherin, N-cadherin, Snail and Twist in CAL27 cells stably expressing either control shRNA or GRIM-19 shRNA. GRIM-19 knockdown led to decreased E-cadherin mRNA expression and had little effect on mRNA levels of N-cadherin, Snail and Twist in CAL27 cells (Figure 4K).

To determine the effects of GRIM-19 gene knockdown on *in vivo* tumorigenicity, CAL27 cells stably expressing either control shRNA or GRIM-19 shRNA were subcutaneously injected into 10 nude mice and examined for tumor formation. Each mouse was injected with a control inoculation in one flank and a GRIM-19 shRNA inoculation in the other flank, to ensure tumor comparisons were controlled for each mouse. Tumor growth was monitored every 3 days, and tumors were excised and weighed at 21 days post-injection. The results demonstrated that GRIM-19 knockdown cells formed larger tumors *in vivo* compared to control cells (Figure 4L, M). Western blot and quantitative PCR analyses of protein and RNA, respectively, from the excised tumors confirmed maintenance of both the GRIM-19 knockdown and lower p53 activation phenotypes (Figure 4N, 4O and Figure S3).

## DISCUSSION

In this study, we demonstrated a genome-wide methylation profile and screened for differentially methylated CGIs and candidate genes in tissue samples from HNSCC and normal oral mucosa. Methylated CGIs were widespread in both the gene-related regions and genomic unknown regions in tumor and control tissues. For the first time, we identified and confirmed a novel promoter hypermethylation in GRIM-19 in patients with HNSCC. GRIM-19 hypermethylation may be useful as a marker for HNSCC with 90% sensitivity and 77% specificity. We observed that decreased GRIM-19 expression not only altered the p53- and HIF-1α signaling pathways, but also promoted aerobic glycolysis and cell proliferation in HNSCC cells.

Annotation of the identified methylated CGIs revealed a significant clustering of DNA methylation in the genome in both HNSCC and normal mucosa. We identified numerous promoter regions that were differentially hypermethylated in HNSCC and normal mucosa. We identified 35 candidate genes that had highly hypermethylated promoters in HNSCC. Eight of these genes, including SOX1 [17–20], WIF1 [21, 22], ACRC [23], ADCY5 [24], BCAP31 [25], NKX6-2 [26], PLK5P [27], and PRSS21 [28], have been reported to be hypermethylated in cancers. Two of the highly hypermethylated candidates, namely GRIM-19 [11–14] and ABCD1 [32, 33] have been reported to have altered expressions in cancers. The function of ABCD1 is related to very long chain fatty acid (VLCA) metabolism [29]. Down-regulation of ABCD1 expression in colon and renal cancer has been suggested as a failure of peroxisomal biogenesis [32, 33]. The importance of ABCD1 during HNSCC development needs to be explored further. Indeed, several genes, such as LINE-1 [34], CSPG4 [35], and TKTL1 [36] have been reported to be hypomethylated in HNSCC. In the current study, we observed 33 new candidates of hypomethylation including ABCB6, BTG1 and EPB41L4B. Intriguingly, increased expressions of ABCB6, BTG1 and EPB41L4B were found in glioma [37], thyroid carcinoma [38] and prostate cancer [39], respectively, but the underlying mechanism stays unclear. Our data indicated that hypomethylation might be associated with the increased expressions of these genes.

We identified GRIM-19 as a novel promoter hypermethylated gene. Because IFN/RA-induced apoptosis lacks either a functional p53 and/or caspase-3 mediating pathway, Hophmann *et al*. isolated GRIM-19 [40], which augmented the effect of IFN/RA-induced apoptosis [9]. Additionally, 93% of RCCs lost GRIM-19 expression [15]. GRIM-19 expression level was negatively correlated with the stage of the primary lesion in lung cancer [11]. Consistently, we observed a tendency toward lower GRIM-19 expression in patients with HNSCC. Promoter hypermethylation provides a possible underlying mechanism for the down-regulation of GRIM-19 expression in HNSCC.

The presence of methylation in healthy subjects has been detected in previous studies [41], and those data reflect chronic environmental exposures, including smoking, physical activity, diet, or carcinogens. Monozygotic twins are epigenetically indistinguishable during the early years of life, but older twins exhibit remarkable differences in their overall content and genomic distribution of DNA methylation [42]. Profiling of young and old murine hematopoietic stem cells (HSCs) has demonstrated that aged HSCs exhibit broader H3K4me3 peaks across HSC identity and have increased DNA methylation at transcription factor binding sites associated with differentiation-promoting genes [43]. We observed significantly different GRIM-19 hypermethylation between young and elderly HNSCC patients as well as between young and elderly controls. Collectively, age-related changes in DNA methylation may partially underlie the increased cancer risk in the elderly [44].

High rates of aerobic glycolysis or the Warburg effect provides HNSCC with advantages in bioenergetics and biosynthesis [45]. Interestingly, we observed that GRIM-19 expression positively or negatively correlated with oxygen consumption and glycolytic activity in HNSCC cell lines. As a component of complex I in the electron transport system, GRIM-19 is important for regulating the TCA cycle activity [46]. In addition, GRIM-19 has been reported to negatively regulate HIF-1α level in a Stat3-dependent manner in glioblastoma cells [47]. Both HIF-1α and Stat3 promote aerobic glycolysis and downregulate oxidative phosphorylation in cancer cells [48], suggesting that GRIM-19 may downregulate aerobic glycolysis by reducing HIF-1α and Stat3 levels and activities. To determine if altered GRIM-19 levels affect metabolic activities in HNSCC cells, we ectopically expressed GRIM-19 in GRIM-19 low-expressing JHU-028 cells and suppressed GRIM-19 expression in GRIM-19 high-expressing CAL27 cells. As expected, JHU-028 cells stably expressing HA-GRIM-19 displayed increased oxygen consumption as well as reduced glucose uptake and lactate production. Conversely, CAL27 cells stably expressing GRIM-19 shRNA displayed decreased oxygen consumption as well as increased glucose uptake and lactate production. These data indicate that low levels of GRIM-19 contribute to aerobic glycolysis, namely the Warburg effect, in HNSCC cells. We also observed that GRIM-19 overexpression inhibited Stat3 and HIF-1α activities and that GRIM-19 knockdown promoted Stat3 and HIF-1α activities, accompanied by decreased and increased aerobic glycolysis and proliferation, respectively, in HNSCC cells. Our findings indicate that GRIM-19 reprograms metabolic activity of HNSCC cells possibly by regulating the Stat3 and HIF-1α pathways.

Interestingly, treating JHU-028 cells with 5-AZA increased GRIM-19 mRNA expression, decreased cell proliferation and promoted oxygen consumption, which is similar to what we observed in JHU-028 cells overexpressing GRIM-19. We also observed higher glycolytic activity in JHU-028 cells treated with 5-AZA, probably due to the demethylation effect of 5-AZA on other genes directly or indirectly regulating glycolysis. For example, it has been reported that expression of hexokinase and lactate dehydrogenase can be increased by 5-AZA treatment [49].

Increased and decreased p53 activity may partially contribute to the lower and higher rates of cell proliferation in HNSCC cells transfected with GRIM-19 cDNA or shRNA. We determined p53 activity by examining the level of phosphor-p53 (Ser15) and mRNA expression of p53 target genes such as p21, TIGAR and Mdm2. Although Mdm2 protein decreases p53 levels by promoting its protein degradation, increased Mdm2 mRNA expression does not necessarily lead to higher Mdm2 protein levels. Therefore, it is not hard to understand the presence of high or low levels of both phosphor-p53 (Ser15) and Mdm2 mRNA in our findings. The mechanism by which p53 activity is regulated by the level of GRIM-19 remains unknown and should to be elucidated in the future.

Stat3 promotes EMT by regulating many key factors in the process. We found that the mRNA expression of E-cadherin increased or decreased when GRIM-19 was ectopically expressed or suppressed by shRNA in HNSCC cells. Whether GRIM-19 plays a role in EMT by regulating E-cadherin mRNA expression *via* Stat3 is worthy of further investigation.

## MATERIALS AND METHODS

### Samples

Tissue samples from HNSCC and the oral mucosa of healthy subjects were obtained from the Department of Oral Mucosal Diseases and the Department of Oral Maxilloficial Surgery of Shanghai 9^th^ People's Hospital, Shanghai Jiao Tong University School of Medicine. The study was approved by the institutional review board and signed informed consent was obtained from participants. Tissue samples obtained from 3 patients with HNSCC and oral mucosa from 3 healthy subjects were used for a methylated DNA immunoprecipitation (MeDIP) array experiment. Another cohort of 30 HNSCCs and 31 healthy subjects were used for verification. The features of enrolled subjects are summarized in Table 1 and Table S3.

### Cell culture

The JHU-011, JHU-022, JHU-028, and Fadu human HNSCC lines were gifts from Dr. Califano at the Johns Hopkins University and were cultured in RPMI 1640 (Invitrogen, Carlsbad, CA, USA) supplemented with 10% FBS, 2 mmol/l L-glutamine, 100 unit /ml penicillin, and 100 g/ml streptomycin at 37ºC in humidified 5% CO_2_ atmosphere. The CAL27 cell line was obtained from the American Type Culture Collection (ATCC, Rockville, MA, USA) and cultured in DMEM with 10% fetal calf serum at 37°C and 5% CO_2_.

### MeDIP array and data analysis

MeDIP was performed by KangChen Bio-tech (Shanghai, China) using the protocol suggested by NimbleGen (Roche-NimbleGen, Madison, WI, USA) [50]. The NimbleGen HG18 CpG Promoter Array, is a single array design including all well-characterized RefSeq promoter regions and all known CpG Islands annotated by UCSC, and it has 385,000 probes that cover all 24,659 gene promoters reported in the Human RefSeq and 28,226 CGIs annotated in the UCSC genome browser. Array was scanned using the Axon GenePix 4000B microarray scanner and data were extracted using the NimbleScan and SignalMap software. A candidate gene interaction pathway analysis was performed using KEGG orthology. The raw data has been deposited in Experiment-Compliant Database (GEO). The accession number is GSE58630.

### Genomic methylation-specific PCR, bisulfite sequencing, and real-time methylation-specific PCR

Genomic DNA was isolated using a Qiagen DNeasy Tissue Kit (Qiagen, Düsseldorf, Germany). Bisulfite treatment was performed using the EpiTect Bisulfite kit (Qiagen, Düsseldorf, Germany) according to the manufacturer's instructions. Bisulfite-treated DNA 10 ng was used for PCR (BSP). Methylation-specific PCR (MSP) and unmethylation-specific PCR (USP) were performed using a Technet-512 (Technet, Staffordshire, UK). The MSP reaction included an initial incubation at 95°C for 5 min, followed by 40 cycles of 95°C for 30 seconds, 58°C for 20 seconds and 72°C for 20 seconds, followed by one cycle of 72°C for 10 min. For bisulfite sequencing, the PCR product was TOPO-cloned into the pCR4 vector (Invitrogen Life Technologies, CA, USA) and 5 positive clones were selected for sequencing. The real-time methylation-specific PCR (QMSP) reaction included an initial incubation at 95°C for 5 min, followed by 40 cycles of 95°C for 10 seconds, 58°C for 10 seconds, 72°C for 20 seconds, and 81°C for 1 second. SYBR Green I (Sigma-Aldrich, St.Louis, MO, USA) was added following the manufacturer's instructions. Bisulfite-treated DNA 10 ng was used in QMSP with a LightCycler480 (Roche, Basel, Switzerland). All primers are shown in Table S4.

### 5-Aza-2′-deoxycytidine treatment

For demethylation analysis, 1.5 × 10^5^ CAL27 cells were seeded, incubated for 24 h and treated with 2.5, 5, and 10 μM 5-aza-2-deoxycytidine (5-AZA, Sigma, St Louis, MO, USA). The media containing 5-AZA was changed every 24 h for 3 days and treated cells were collected at 0, 24, and 48 h. Similarly, JHU-028 cells were treated with control or 5 μM 5-AZA for 24, 48, 72 and 96 h, respectively and assayed for GRIM-19 mRNA levels using real time PCR analysis. Oxygen consumption, glucose uptake and lactate production were measured at 48 h after 5-AZA treatment.

### RNA extraction, cDNA synthesis and real-time PCR

Total RNA was extracted using TRIzol (Invitrogen Life Technologies, Carlsbad, CA, USA) according to the manufacturer's instructions. Total RNA was reverse transcribed into cDNA using the PrimeScript™ RT reagent kit (Takara Bio Inc., Shiga, Japan). The SYBR® green Premix Ex Taq TM kit (Takara Bio Inc., Shiga, Japan) was used for real-time PCR (QPCR) analysis which was performed using an ABI 7500 fast Sequence Detector (Applied Biosystems, Carlsbad, CA, USA). All primers are listed in Table S4.

### Metabolic assays

To measure oxygen consumption, 80% confluent cells in 10 cm dishes were trypsinized and resuspended in 3 ml of medium. Oxygen consumption was measured using an Oxytherm system (Hansatech, Kings Lynn, UK).

To measure glucose uptake and lactate production, cells were plated in 6-well plates. 48 hours after plating or treatment, culture medium was collected and investigated using the glucose assay kit (Shanghai Rongsheng Biotech, Shanghai, China) and lactate assay kit (Sigma, St Louis, MO, USA) following manufactures' instructions.

### Western blotting analysis

Total protein extracts were obtained using RIPA lysis buffer (Sigma, St Louis, MO, USA). Protein concentration was measured using a BCA Protein Assay kit (Pierce, Rockford, IL, USA). Samples were subsequently resolved in 7.5 and 15% SDS-PAGE gels. Proteins were transferred to Immuno-Blot PVDF Membrane (Bio-Rad, Hercules, CA, USA) and the membranes were blocked using 5% non-fat milk in Tris-buffered saline with Tween-20. The membrane was incubated with primary antibodies followed by incubation with secondary peroxidase labeled anti-rabbit or anti-mouse antibodies (Santa Cruz Biotechnology, Inc., Santa Cruz, CA, USA). The protein signals were detected using an enhanced chemiluminescent solution (Millipore, Boston, MA, USA). Primary antibodies used are listed in the Supplementary Materials.

### Cell proliferation

Cells were plated in 6-well plates with a density of 8000 cells/well. Cell numbers were counted at day 1, 2, 3 and 4 after plating.

### ShRNA and cDNA transfection

The GRIM-19 shRNA construct was obtained from Open Biosystems (Huntsville, AL, USA) with the following shRNA sequence (sense): 5′-GCGCCTACAAATCGAGGACTT-3′. The cDNA encoding the complete coding region of human GRIM-19 cDNA was obtained from GeneBank (NM 015965.6) and then subcloned into the lentiviral vector.

The shRNA and cDNA were transfected into 293T cells with the packaging plasmids, PMD2G and PSPX2, using Lipofectamine 2000 (Invitrogen Life Technologies, Carlsbad, CA, USA) according to the manufacturer's instructions. Viral supernatant was collected after 48 h. JHU-028 and CAL27 cells were plated in 10 cm dishes, and the medium was removed after 24 h. Viral supernatant (5 ml) and 5 ml of fresh medium were then added to the cells, and the medium was changed to fresh medium after 24 h. JHU-028 stably transfected cells were selected using flow cytometry, and CAL27 stably transfected cells were selected using 2 μg/ml puromycin.

### Xenograft experiments

Xenograft experiments were performed as previously described [51]. Briefly, 1 × 10^7^ CAL27 cells resuspended in 200 μl PBS were subcutaneously injected into the flanks of athymic nude male mice. 10 mice numbered from #1 to #10 were injected. Each mouse was injected in two flanks including a control and a GRIM-19 shRNA inoculation. Tumor size was monitored every 3 days. 21 days after injection, tumors were excised, weighed and assayed for mRNA and protein expression.

### Statistical analysis

Statistical analysis was performed using Prism 5 (GraphPad Software, San Diego, CA, USA) and SPSS (IBM, Armonk, NY, USA). Mann-Whitney test was used to compare gene methylation or expression levels between HNSCC and control samples. The overall survival and disease-free survival of patients with HNSCC were analyzed using the Log-Rank test. An ROC analysis was used to measure the separation of clinical settings. Youden's index was used to provide the optimal a cutoff for sensitivity and specificity for clinical application. All reported *P* values were 2-sided and *P* < 0.05 was considered statistically significant.

## SUPPLEMENTARY METHOD, FIGURES AND TABLES


