# Supplementary material for: Decreased expression of GRIM-19 by DNA hypermethylation promotes aerobic glycolysis and cell proliferation in head and neck squamous cell carcinoma
Source: Oncotarget. 2014 Dec 23;6(1):101–15. doi: 10.18632/oncotarget.2684 (PMC4381581; doi:10.18632/oncotarget.2684)
Supplement: Supplementary file 1 [file oncotarget-06-101-s001.pdf]

# SUPPLEMENTARY METHOD, FIGURES AND TABLES

## Antibodies

Anti-GRIM-19 (eBioscience, USA), anti-tubulin (Sigma, USA), anti-parp (Invitrogen, USA), anti-p53, anti-phospho-p53, anti-p21, anti-HIF-1α, anti-Stat3, anti-phospho-Stat3 (Cell Signaling Technology, USA).

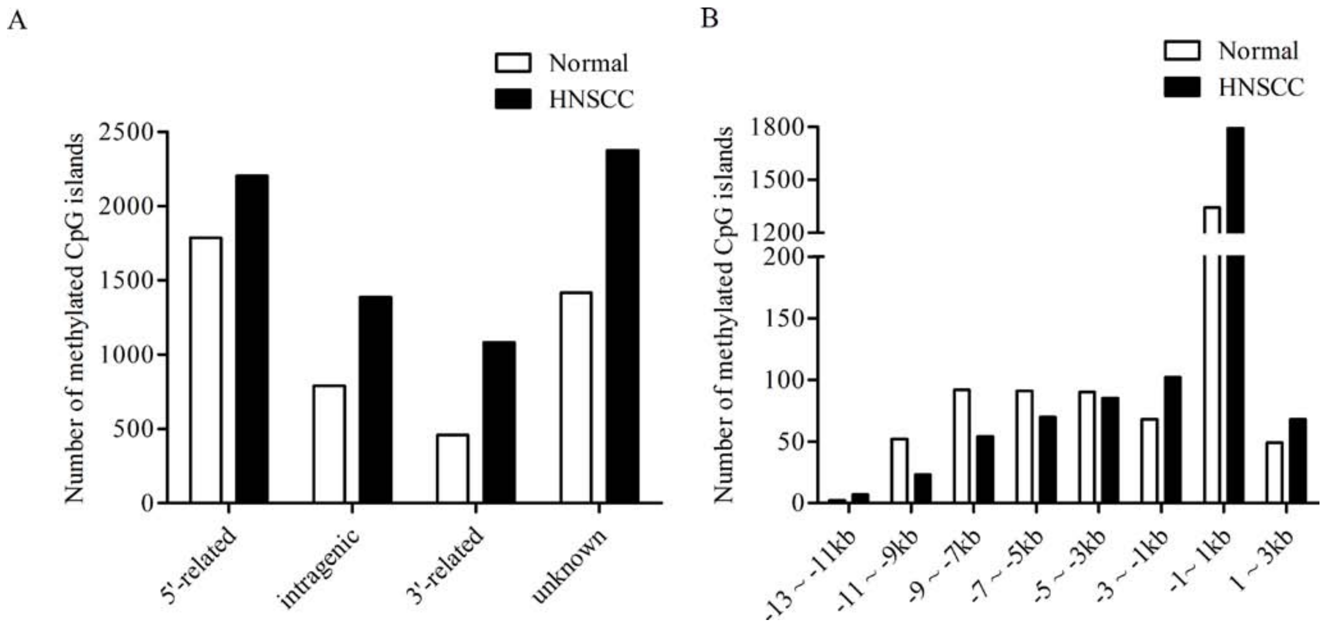


**Supplementary Figure S1: Genome-wide DNA methylation profile for altered DNA methylation.** **(A)** Distribution of methylated CGIs in genome of HNSCC and control samples. **(B)** Distribution of methylated CGIs in 5′-gene related region of HNSCC and control samples.

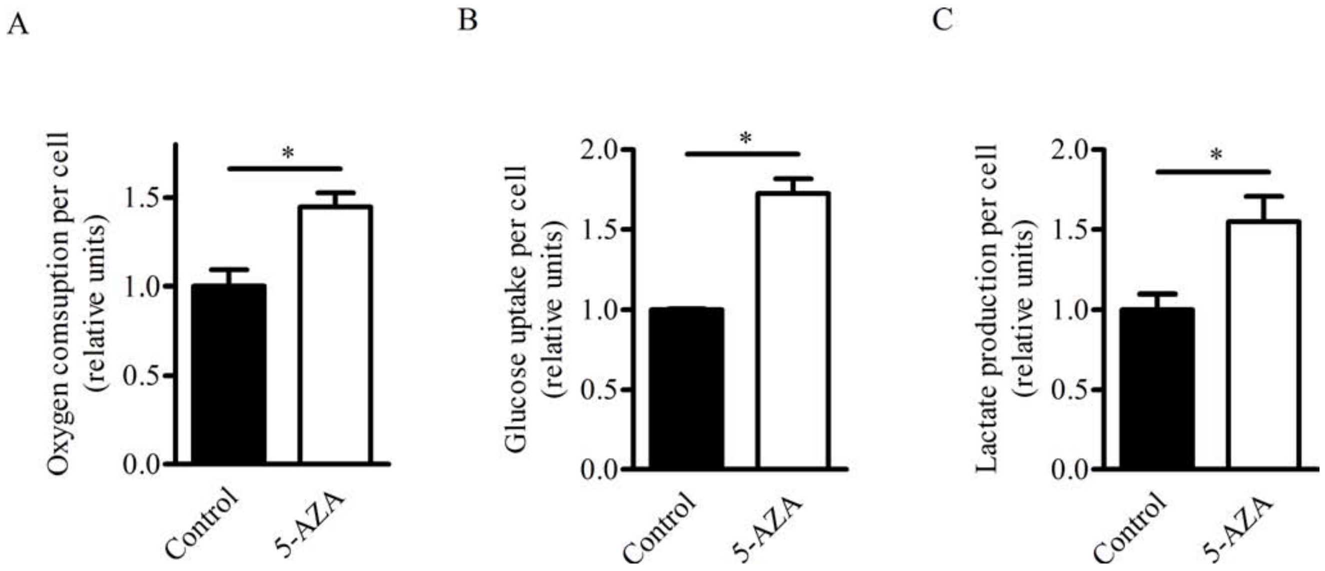


**Supplementary Figure S2: 5-AZA treatment alters metabolic activities in JHU-028 cells.** **(A)** Oxygen consumption, **(B)** glucose uptake, and **(C)** lactate production of JHU-028 cells treated with vehicle control or 5 μM 5-AZA for 48 hours. The data in (A)-(C) were normalized by cell number.

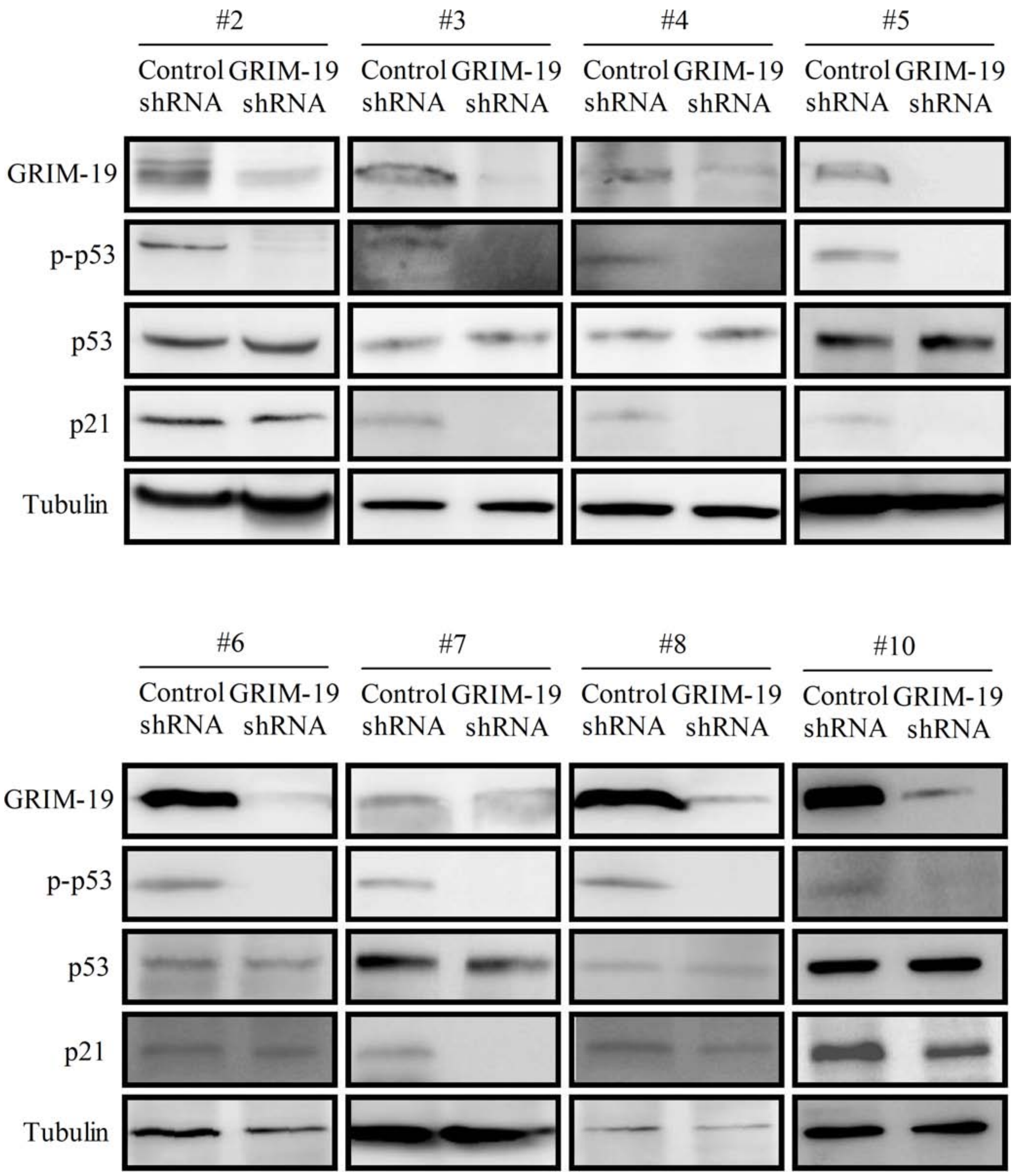


**Supplementary Figure S3: Western blot analysis of protein extracts of tumors formed in #2, #3, #4, #5, #6, #7, #8 and #10 nude mice 21 days after injection of CAL27 cells stably expressing either control or GRIM-19 shRNA.** We failed to exact protein from tumors formed in the #9 mouse. Tubulin blots serve as loading controls.

**Supplementary Table S1. Hypermethylated candidate genes in HNSCC**

| Gene | Methylation reported in tumor | Decreased expression in tumor | KEGG Pathway |
|---|---|---|---|
| ACOT7 | | | |
| ACTL7B | | | |
| AFF2 | | | |
| AGAP2 | | | |
| AMIGO3 | | | |
| ANKRD30A | | | |
| ARHGEF10 | | | |
| ARSB | | | |
| B3GNT1 | | | |
| BCL2L11 | salivary gland adenoid cystic carcinoma, CLL | | |
| BCORL1 | | | |
| BOK | | | |
| BOLA2B | | | |
| BRSK2 | | | |
| C14orf104 | | | |
| C16orf14 | | | |
| C16orf7 | | | |
| C17orf98 | | | |
| C18orf8 | | | |
| C21orf81 | | | |
| C22orf24 | | | |
| C2orf84 | | | |
| C3orf70 | | | |
| C7orf42 | | | |
| CBFA2T3 | | | |
| CCDC144B | | | |
| CDH23 | | | |
| CGNL1 | | | |
| CHMP6 | | | |
| CLK2P | | | |
| COL5A1 | | | |
| CPNE7 | ALL | ALL | |
| CPSF3L | | | |
| CTCF | Oral CA | | |
| CUX1 | | ALL | |

(*Continued*)

| Gene | Methylation reported in tumor | Decreased expression in tumor | KEGG Pathway |
|---|---|---|---|
| CXCL12 | Breast CA, gastric CA, NSCLC | Breast CA, Colon CA | Axon guidance, Cytokine-cytokine receptor interaction, Chemokine signaling pathway |
| DDAH2 | Oral CA | | |
| DOC2B | Cervical CA | Cervical CA | |
| DUSP9 | | Renal CA | |
| EFNB1 | ALL | ALL | Axon guidance |
| EGR3 | Breast CA | Gastric CA | |
| EIF4E3 | | | |
| ELF4 | | | |
| EMD | | Endometrial CA | Dilated cardiomyopathy |
| EVX1 | Prostate CA, NSCLC | Prostate CA | |
| F8 | | | |
| F8A3 | | | |
| FAM108C1 | | | |
| FAM48B1 | | | |
| FLJ35220 | | | |
| FLJ40330 | | | |
| FLJ44054 | | | |
| FOXI3 | | | |
| FRAT1 | | | Wnt signaling pathway |
| FRMD1 | | | |
| FTHL17 | | | |
| FTMT | | | |
| GAL | | | |
| GAS1 | | Gastric CA, Colon CA | |
| GATA5 | Lung CA, Oral CA, ovarian CA | Renal CA, Colon CA | |
| GLTPD1 | | | |
| GPC1 | | | |
| GPR135 | | | |
| GPR45 | | | |
| GPR75 | | | |
| GRAMD4 | | | |
| GTF2I | | | |
| HAGHL | | | |
| HCFC1 | | | |
| HDLBP | | | |

(*Continued*)

| Gene | Methylation reported in tumor | Decreased expression in tumor | KEGG Pathway |
|---|---|---|---|
| HEATR2 | | | |
| HES5 | neuroblastoma tumor, alveolar rhabdomyosarcoma, ALL | | |
| HHEX | | | |
| HMGA1 | Tumor cells | | |
| HSF2BP | | | |
| IGFBPL1 | Breast CA | Breast CA | |
| INSL6 | | | |
| JAG1 | ALL | Endometrial CA | |
| KCNAB3 | | | |
| KCTD1 | | | |
| KCTD11 | Medulloblastoma | Medulloblastoma | |
| KDM3B | | | |
| KIF12 | | | |
| KRTCAP3 | | | |
| LAGE3 | | | |
| LOC100130776 | | | |
| LOC407835 | | | |
| LOC442459 | | | |
| LOC729627 | | | |
| LONRF2 | | | |
| LPAR1 | | | Gap junction |
| LRIG1 | | Bladder CA, Cervical CA | |
| LRRFIP1 | | | |
| LSP1 | | | |
| LY6K | | | |
| MAP7D2 | | | |
| MECP2 | | | |
| MEST | Cervical CA | | |
| MFSD6 | | | |
| MOV10L1 | | | |
| MPV17L | | | Peroxisome |
| MRPL40 | | | |
| NAT8L | | | |
| NIN | | | |
| NKX2-4 | salivary gland adenoid cystic carcinoma | | |

(*Continued*)

| Gene | Methylation reported in tumor | Decreased expression in tumor | KEGG Pathway |
|---|---|---|---|
| NPTX1 | colon CA, cervical CA, Pancreatic CA | Pancreatic CA | |
| NR0B1 | Lung adenocarcinoma | | |
| NR1D2 | | | |
| NUDT11 | | | |
| NUP210 | | | |
| OTUD1 | | | |
| OTUD6A | | | |
| OXCT2 | | | |
| PAFAH1B1 | | | |
| PGRMC1 | | | |
| PIP5K1C | | | Fc gamma R-mediated phagocytosis, Regulation of actin |
| PLEKHA2 | | | |
| PLXNA1 | | | Axon guidance |
| PLXNC1 | | ALL | Axon guidance |
| PNMA6A | | | |
| POLR2I | | | |
| POM121L9P | | | |
| PPP1R2P9 | | | |
| PPP1R9A | | | |
| PREB | | | |
| PRKACG | | | Wnt signaling pathway, Gap junction, Dilated cardiomyopathy |
| PXK | | | |
| QRICH2 | | | |
| RCAN1 | | | |
| RIMKLA | | | |
| RNF19B | | | |
| ROCK2 | | | Axon guidance, Wnt signaling pathway, TGF-beta signaling pathway, Vascular smooth muscle contraction |
| RORA | Gastric CA | Colon CA | |
| RRP1B | | | |
| SCXB | | | |
| 6-Sep | | | |
| SH3GLP3 | | | |

(*Continued*)

| Gene | Methylation reported in tumor | Decreased expression in tumor | KEGG Pathway |
|---|---|---|---|
| SH3RF1 | | | |
| SLC25A2 | | | |
| SLC29A4 | | | |
| SLC30A1 | | | |
| SMAD3 | | HNSCC | Wnt signaling pathway, Pathways in cancer, Endocytosis, TGF-beta signaling pathway |
| SNAP91 | | | |
| SOX30 | | | |
| SOX9 | Gastric CA, Bladder CA | Pancreatic CA | |
| TACC2 | | | |
| TAF1 | | | |
| TBCB | | | |
| TBL1X | | | Wnt signaling pathway |
| TDRD6 | | | |
| TFE3 | | Ovarian CA | |
| TMEM185A | | Ovarian CA | |
| TMEM187 | | | |
| TMEM47 | | | |
| TMEM65 | | | |
| TRPC3 | | | |
| TSP50 | | | |
| TUBA3D | | | Gap junction, Pathogenic Escherichia coli infection |
| TUBA3E | | | Gap junction, Pathogenic Escherichia coli infection |
| UNC5D | Renal CA | Renal CA | Axon guidance |
| UPB1 | | | |
| WDR21B | | | |
| WDR21C | | | |
| WDR88 | | | |
| XKR6 | | | |
| YWHAH | | | |
| YY2 | | | |
| ZC3HAV1L | | | |
| ZIC3 | | | |
| ZNF639 | | | |
| ZNF718 | | | |
| ZNF777 | | | |
| ZNF786 | | | |

**Supplementary Table S2. Highly hypomethylated candidate genes in HNSCC**

| Genes | Biological Process |
|---|---|
| RBMY1A1, RBMY1F, RBMY1B | RNA splicing |
| | mRNA processing |
| | spermatogenesis |
| RBMY1A1, RBMY1F, RBMY1B, LSM11 | mRNA processing |
| RBMY1A1, RBMY1F, RBMY1B, TSPY3 | spermatogenesis |
| BTG1, SAP130, ZNF783, VGLL4, WHSC1 | regulation of transcription, DNA-dependent |

**Supplementary Table S3. Clinical information of HNSCC and control**

| ID | Diagnosis | Age | Gender | Smoking | Alcohol | Stage TNM[a] | Note | Tumor Site |
|---|---|---|---|---|---|---|---|---|
| 1 | Normal | 25 | M | NA | Past | | DNA | |
| 2 | Normal | 42 | F | Past | Past | | DNA | |
| 3 | Normal | 53 | F | Never | Past | | DNA | |
| 4 | Normal | 40 | M | Never | Never | | DNA | |
| 5 | Normal | 25 | M | NA | Never | | DNA | |
| 6 | Normal | 34 | F | Current | Past | | DNA | |
| 7 | Normal | 37 | M | Never | Past | | DNA | |
| 8 | Normal | 41 | M | NA | Past | | DNA | |
| 9 | Normal | 52 | F | Current | Past | | DNA | |
| 10 | Normal | 36 | M | Never | Past | | DNA | |
| 11 | Normal | 42 | F | Current | Past | | DNA | |
| 12 | Normal | 31 | M | NA | Never | | DNA | |
| 13 | Normal | 27 | F | NA | Never | | DNA | |
| 14 | Normal | 24 | M | NA | Never | | DNA | |
| 15 | Normal | 34 | M | NA | Never | | DNA | |
| 16 | Normal | 28 | F | NA | Past | | DNA | |
| 17 | Normal | 23 | F | NA | Never | | DNA | |
| 18 | Normal | 34 | F | Never | Past | | DNA | |
| 19 | Normal | 35 | M | NA | Never | | DNA | |
| 20 | Normal | 30 | M | NA | Never | | DNA | |
| 47 | HNSCC | 49 | M | Never | Never | NA | DNA/RNA | Tongue |
| 51 | HNSCC | 48 | F | Never | Never | T1N0M0 | DNA/RNA | Tongue |
| 52 | HNSCC | 72 | M | Past | Never | T2N0M0 | DNA | Tongue |
| 53 | HNSCC | 51 | M | Current | Current | T2N0M0 | DNA/RNA | Floor of mouth |
| 54 | HNSCC | 28 | F | Never | Never | T3N0M0 | DNA/RNA | Tongue |
| 55 | HNSCC | 67 | F | Never | Never | T3N0M0 | DNA/RNA | Gingiva |
| 56 | HNSCC | 77 | F | Never | Never | T1N1M0 | DNA/RNA | Retromolar area |
| 57 | HNSCC | 41 | F | Never | Never | T2N2M0 | DNA/RNA | Tongue |
| 58 | HNSCC | 59 | F | Never | Never | T1N0M0 | DNA/RNA | Tongue |
| 59 | HNSCC | 32 | M | Never | Never | T4N0M0 | DNA/RNA | Tongue |
| 60 | HNSCC | 38 | F | Never | Never | T1N0M0 | DNA/RNA | Tongue |
| 61 | HNSCC | 63 | M | Current | Never | T2N0M0 | DNA/RNA | Gingiva |
| 62 | HNSCC | 42 | M | Never | Current | T1N0M0 | DNA/RNA | Tongue |
| 63 | HNSCC | 60 | M | Current | Never | T1N1M0 | DNA/RNA | Oropharynx |
| 64 | HNSCC | 36 | M | Current | Past | T3N1M0 | DNA/RNA | Gingiva |

(*Continued*)

| ID | Diagnosis | Age | Gender | Smoking | Alcohol | Stage TNM[a] | Note | Tumor Site |
|---|---|---|---|---|---|---|---|---|
| 66 | HNSCC | 47 | F | Never | Current | T2N2M0 | DNA/RNA | Gingiva |
| 67 | HNSCC | 38 | M | Current | Current | T4N2M0 | DNA/RNA | Floor of mouth |
| 68 | HNSCC | 55 | M | Current | Current | T2N0M0 | DNA/RNA | Tongue |
| 69 | HNSCC | 59 | F | Never | Never | T3N0M0 | DNA/RNA | Buccal |
| 70 | HNSCC | 47 | F | Never | Never | T3N1M0 | DNA/RNA | Tongue |
| 71 | HNSCC | 57 | F | Never | Never | T2N0M0 | DNA/RNA | Gingiva |
| 72 | HNSCC | 58 | F | Never | Current | T3N0M0 | DNA/RNA | Tongue |
| 73 | HNSCC | 60 | M | Current | Current | T3N0M0 | DNA/RNA | Tongue |
| 74 | HNSCC | 57 | F | Never | Never | T3N0M0 | DNA/RNA | Gingiva |
| 75 | HNSCC | 75 | F | Never | Never | T3N1M0 | DNA/RNA | Gingiva |
| 76 | HNSCC | 58 | F | Never | Never | T3N0M0 | DNA/RNA | Gingiva |
| 77 | HNSCC | 53 | F | Never | Never | NA | DNA/RNA | Tongue |
| 78 | HNSCC | 48 | M | Never | Current | T2N0M0 | DNA/RNA | Tongue |
| 79 | HNSCC | 79 | M | Past | Current | T4N0M0 | DNA/RNA | Gingiva |
| 80 | HNSCC | 78 | F | Never | Never | T2N1M0 | DNA/RNA | Gingiva |
| 82 | Normal | 51 | F | NA | NA | | DNA/RNA | |
| 84 | Normal | 53 | F | NA | NA | | DNA/RNA | |
| 86 | Normal | 61 | F | Never | Never | | DNA/RNA | |
| 89 | Normal | 69 | F | Never | Never | | DNA/RNA | |
| 90 | Normal | 61 | F | Never | Never | | DNA/RNA | |
| 92 | Normal | 62 | F | Never | Never | | DNA/RNA | |
| 93 | Normal | 52 | F | Never | Never | | DNA/RNA | |
| 94 | Normal | 61 | M | Never | Never | | DNA/RNA | |
| 95 | Normal | 56 | M | NA | NA | | DNA/RNA | |
| 97 | Normal | 54 | F | NA | NA | | DNA/RNA | |
| 98 | Normal | 59 | F | NA | NA | | DNA/RNA | |
| 99 | HNSCC | 65 | F | Never | Never | T2N1M0 | RNA | Buccal |
| 101 | Normal | 51 | F | Never | Never | | RNA | |
| 102 | Normal | 59 | M | NA | NA | | RNA | |
| 103 | Normal | 68 | F | Never | NA | | RNA | |
| 104 | Normal | 50 | M | Never | Never | | RNA | |
| 105 | Normal | 61 | M | Never | Never | | RNA | |
| 106 | Normal | 68 | F | Never | Never | | RNA | |
| 107 | Normal | 60 | M | NA | NA | | RNA | |
| 108 | Normal | 63 | M | NA | Never | | RNA | |
| 109 | Normal | 61 | M | Never | NA | | RNA | |

(*Continued*)

| ID | Diagnosis | Age | Gender | Smoking | Alcohol | Stage TNM[a] | Note | Tumor Site |
|---|---|---|---|---|---|---|---|---|
| 110 | Normal | 25 | M | Never | Never | | Array | |
| 111 | Normal | 22 | M | Never | Never | | Array | |
| 112 | Normal | 24 | M | Never | Never | | Array | |
| 113 | HNSCC | 70 | F | Never | Never | | Array | Gingiva |
| 114 | HNSCC | 78 | F | Never | Never | | Array | Gingiva |
| 115 | HNSCC | 60 | F | Never | Never | | Array | Tongue |

Note: HNSCC, Head and neck squamous cell carcinoma; NA, data not available;
[a]Union for International Cancer Control; T, tumor size; N, lymph node; M, Metastasis; DNA, methylation test; DNA/RNA, both DNA methylation and RNA expression test; RNA, RNA expression test.

**Supplementary Table S4. Primer sequence**

| Primer | Sequence | Experiment |
|---|---|---|
| GRIM19-MSP-F | 5′-AAACGTCGTACTCTACGTCATA-3′ | MSP |
| GRIM19-MSP-R | 5′-TGTAGCGTTCGGATAGTTC-3′ | MSP |
| GRIM19-uMSP-F | 5′-TTTTGTAGTGTTTGGATAGTTT-3′ | MSP |
| GRIM19-uMSP-R | 5′-AAACATCATACTCTACATCATAATC-3′ | MSP |
| GRIM19-BSP-F | 5′ -AGGGAAAAAGYGTATGTGTAGT-3′ | BSP |
| GRIM19-BSP-R | 5′ -CCACCRACCTACTACAAACC-3′ | BSP |
| GRIM19-QRT-F1 | 5′-AGATGCTTCGGGAGAACCTG-3′ | QPCR |
| GRIM19-QRT-R1 | 5′-GCATTTATTCCGTCCCAGTG-3′ | QPCR |
| GAPDH-F | 5′-CAGCCTCAAGATCATCAGCA-3′ | QPCR |
| GAPDH-R | 5′-ACAGTCTTCTGGGTGGCAGT-3′ | QPCR |
| GRIM19-QMSP-F | 5′-AAACGTCGTACTCTACGTCATA-3′ | QMSP |
| GRIM19-QMSP-R | 5′-TGTAGCGTTCGGATAGTTC-3′ | QMSP |
| ACTB-QMSP-F | 5′-TGGTGATGGAGGAGGTTTAGTAAGT-3′ | QMSP |
| ACTB-QMSP-R | 5′-AACCAATAAAACCTACTCCTCCCTTAA-3′ | QMSP |
| GRIM19-QRT-F2 | 5′-GCGTCAAAGGTGAAGCAG-3′ | QPCR |
| GRIM19-QRT-R2 | 5′-CTCCTCCTCCAGGTTCTCC-3′ | QPCR |
| HK2-F | 5′-TCCGTAGTGGGAAAAAGAGAA-3′ | QPCR |
| HK2-R | 5′-GACAATGTGATCAAACAGCTC-3′ | QPCR |
| PFK1-F | 5′-GGTGTACAAGCTTCTAGCTC-3′ | QPCR |
| PFK1-R | 5′-CAAGTTTAGAGCCACCTTGG-3′ | QPCR |
| PKM2-F | 5′-CCACTTGCTGTGCCAAATGGA-3′ | QPCR |
| PKM2-R | 5′-GAAGGACTTTACCTTCCAGGA-3′ | QPCR |
| PDK1-F | 5′-GAAGCAGTTCCTGGACTTCG-3′ | QPCR |
| PDK1-R | 5′-ACCAATTGAACGGATGGTGT-3′ | QPCR |
| VEGF-F | 5′-TACCTCCACCATGCCAAGTG-3′ | QPCR |
| VEGF-R | 5′-ATGATTCTGCCCTCCTCCTTC-3′ | QPCR |
| Mdm2-F | 5′-TGGCGTGCCAAGCTTCTCTGT-3′ | QPCR |
| Mdm2-R | 5′-ACCTGAGTCCGATGATTCCTGCT-3′ | QPCR |
| p21-F | 5′-ACTGTGATGCGCTAATGGC-3′ | QPCR |
| p21-R | 5′-ATGGTCTTCCTCTGCTGTCC-3′ | QPCR |
| TIGAR-F | 5′-CAGTGATCTCATGAGGACAAAGCA-3′ | QPCR |
| TIGAR-R | 5′-CCATGGCCCTCAGCTCACTTA-3′ | QPCR |
| Actin-F | 5′-GGACTTCGAGCAAGAGATGG-3′ | QPCR |
| Actin-R | 5′-AGCACTGTGTTGGCGTACAG-3′ | QPCR |
| humanN-cadherin-F | 5′-GGACAGTTCCTGAGGGATCA-3′ | EMT |
| humanN-cadherin-R | 5′-GGATTGCCTTCCATGTCTGT-3′ | EMT |
| humanE-cadherin-F | 5′-CTGAGAACGAGGCTAACG-3′ | EMT |

(*Continued*)

| Primer | Sequence | Experiment |
|---|---|---|
| humanE-cadherin-R | 5′-GTCCACCATCATCATTCAATAT-3′ | EMT |
| humanSnail-F | 5′-GACCACTATGCCGCGCTCTT-3′ | EMT |
| humanSnail-R | 5′-TCGCTGTAGTTAGGCTTCCGATT-3′ | EMT |
| humanTwist-F | 5′-GGAGTCCGCAGTCTTACGAG-3′ | EMT |
| humanTwist-R | 5′-TCTGGAGGACCTGGTAGAGG-3′ | EMT |
